# Acupuncture for combat post-traumatic stress disorder: trial development and methodological approach for a randomized controlled clinical trial

**DOI:** 10.1186/s13063-021-05394-3

**Published:** 2021-09-06

**Authors:** Michael Hollifield, An-Fu Hsiao, Kala Carrick, Andrea Gory Munoz, Teresa Calloway, Karen Cocozza, Besa Smith, Tyler Smith, Tanja Jovanovic, Seth Norrholm, Estate Sokhadze, Christopher Reist

**Affiliations:** 1 Tibor Rubin VA Medical Center, 5109 E. 7th Street, Long Beach, CA 90822 USA; 2 The George Washington University School of Medicine and Health Sciences, Washington, DC, 2300 I Street NW, Washington, DC 20052 USA; 3 University of California at Riverside, 900 University Ave, Riverside, CA 92521 USA; 4 UC Irvine Health Policy Research Institute, 100 Theory, Suite 110, Irvine, CA 92697 USA; 5 Analydata, 3835 Centraloma Drive, San Diego, CA 92107 USA; 6 Department of Psychiatry and Behavioral Neurosciences, Wayne State University School of Medicine, 3901 Chrysler Service Drive, Detroit, MI 48201 USA; 7 University of South Carolina School of Medicine – Greenville, 701 Grove Rd, Greenville, SC 29605 USA

**Keywords:** Post-traumatic stress, Acupuncture, Combat, Veterans, Psychophysiology

## Abstract

**Background:**

Post-traumatic stress disorder (PTSD) is a significant public health problem, affecting approximately 7% of the general population and 13–18% of the combat Veteran population. The first study using acupuncture for PTSD in a civilian population showed large pre- to post-treatment effects for an empirically developed verum protocol, which was equivalent to group cognitive behavior therapy and superior to a wait-list control. The primary objective of this study is to determine both clinical and biological effects of verum acupuncture for combat-related PTSD in treatment-seeking US Veterans.

**Methods:**

This is a two-arm, parallel-group, prospective randomized placebo-controlled clinical trial. The experimental condition is verum acupuncture and the placebo control is sham (minimal) acupuncture in 1-h sessions, twice a week for 12 weeks. Ninety subjects will provide adequate power and will be allocated to group by an adaptive randomization procedure. The primary outcome is change in PTSD symptom severity from pre- to post-treatment. The secondary biological outcome is change from pre- to post-treatment in psychophysiological response, startle by electromyographic (EMG) eyeblink. Assessments will be conducted at pre-, mid-, post-, and 1-month post-treatment, blind to group allocation. Intent-to-treat analyses will be conducted.

**Discussion:**

The study results will be definitive because both clinical and biological outcomes will be assessed and correlated. Issues such as the number needed for recruitment and improvement, use of sham acupuncture, choice of biological measure, and future research need will be discussed.

**Trial registration:**

ClinicalTrials.gov NCT02869646. Registered on 17 August 2016.

**Supplementary Information:**

The online version contains supplementary material available at 10.1186/s13063-021-05394-3.

## Administrative information

**Table:** 

Title	Acupuncture for Combat Post-traumatic Stress Disorder: Trial Development and Methodological Approach for a Randomized Controlled Clinical Trial
Trial Registration	ClinicalTrials.gov ID: NCT02869646, registered 17^th^ August 2016
Trial Registration Dataset	Health conditions: posttraumatic stress disorder. Intervention: verum acupuncture. Placebo: sham acupuncture. Key inclusion criteria: combat veterans with posttraumatic stress disorder (PTSD), age 18-55. Key exclusion criteria: other Serious mental illness, drug or alcohol dependence, previous acupuncture treatment. Study type: interventional, randomized allocation. Target sample size 90. Primary outcome: PTSD symptoms. Key Secondary outcome: startle reflex.
Protocol version	5^th^ June 2020. Version 1
Funding	This work was supported by the VA Merit Award #l01 CX-001416-01 (Project ID: CLNA-02-15F) from the United States (U.S.) Department of Veterans Affairs, Clinical Sciences Research and Development, as well as, supported with resources and the use of facilities at the Tibor Rubin VA Medical Center in Long Beach.
Contributorship	1. Tibor Rubin VA Medical Center, 5109 E. 7^th^ Street, Long Beach, CA 90822, USA - michael.hollifield@va.gov, anfu.hsiao@va.gov, Kala.Carrick-Harkin@va.gov, Andrea.munoz@va.gov, Teresa.Calloway2@va.gov, Karen.Cocozza@va.gov, christopher.reist@va.gov 2. The George Washington University School of Medicine and Health Sciences, Washington, DC, 2300 I Street NW, Washington, DC 20052 michael.hollifield@va.gov 3. UC Irvine Health Policy Research Institute, 100 Theory, Suite 110. Irvine, CA 92697 - ahsiao@uci.edu 4. Analydata, 3835 Centraloma Drive, San Diego, CA. 92107 - besasmith@analydata.com, tylersmith@analydata.com 5. Wayne State University School of Medicine, Department of Psychiatry and Behavioral Neurosciences, 3901 Chrysler Service Drive, Detroit, MI 48201 – tjovanovic@med.wayne.edu, snorrholm@wayne.edu, 6. University of South Carolina School of Medicine - Greenville, 701 Grove Rd., Greenville, SC 29605 - sokhadze@greenvillemed.sc.edu 7. University of California at Irvine, 680 California Ave. Irvine, CA 92697 - creist@uci.edu, All authors contributed to the study concept and study design throughout study. As primary investigators MH and AH conceived the study, led the proposal and protocol development. Acupuncture expertise contributions are by AH, MH, KC, and TC. Design and statistical expertise contributions are by MH, TS, BS and AGM. Psychophysiology expertise is led by TJ, SN, ES, MH, and KC. All authors critically revised and approved the final manuscript.
Sponsor and contact information	Department of Veteran Affairs, Veterans Health Administration, Office of Research and Development, Clinical Science Research and Development; Merit Review Program Michael Hollifield, MD: Michael.hollifield@va.gov
Sponsor and funder	The study sponsor and funder were not involved in the study design, writing of the protocol paper, or the decision to submit the paper for publication.

## Background

Post-traumatic stress disorder (PTSD) is debilitating and characterized by re-experiencing aspects of the incident trauma, avoidance and numbing of trauma reminders, negative alterations in cognition and mood, and hyperarousal [1]. Lifetime prevalence of PTSD in community samples is about 6.8% [2] and as high as 30% in Vietnam Veterans [3] and rape survivors [4]. More recent OEF/OIF/OND Veterans have 13–25% PTSD prevalence, dependent on combat exposure [5, 6].

The first known randomized controlled clinical trial (RCCT) using acupuncture and a traditional Chinese medicine (TCM) approach for PTSD was published in 2007 [7], showing verum acupuncture was superior to a wait-list control and equivalent to group cognitive behavior therapy (CBT). A paper describing protocol development and methodology for that study was published in 2006 [8]. These novel findings led to a broader adoption of acupuncture practice for PTSD in the Veterans Administration Hospitals and across the USA [9] as well as further research regarding the use of acupuncture for PTSD [10–13]. These and other summative findings indicate a complex biology of PTSD including hypothalamic-pituitary-adrenal (HPA) axis dysfunction, autonomic nervous system (ANS) dysfunction, alterations in central nervous system (CNS) processes, and inflammatory dysregulation, all under complex gene x environmental interaction control, and there is a large human and animal literature about biological effects of acupuncture in these systems [14]. Reported research outcomes since the first publication have generally supported the efficacy of acupuncture for PTSD, though methodological limitations inhibit strong supportive consensus [15].

Limitations in early studies were the lack of a placebo (sham) arm, understanding best practices about placebo, use of self-report assessment instruments as primary outcomes, lack of a biological outcome, use of single therapists, and lack of monitoring protocol adherence. In general, scientific discourse about limitations of acupuncture clinical trials persist with the most common concerns being the inability to conduct double-blind trials, the lack of a true placebo, the variability of treatment approaches, the rare assessment of protocol adherence, and the inability to assess non-specific factors on treatment outcome. The current study was designed to address many of these limitations and raise the bar for showing efficacy by having both clinical and biological outcomes and using a sham control.

## Study design and methods

### Design

This is a two-arm, parallel-group, prospective randomized placebo-controlled clinical trial at the Long Beach VA Healthcare System (LBVA), with internal collaboration with The Program for Traumatic Stress’ Novel Therapies Unit, the Integrative Medicine Clinic, the Research Healthcare Group, and the PTSD psychophysiology laboratory, and external collaboration with design and analytic experts from University of California at San Diego and The National University and psychophysiology experts from Wayne State University School of Medicine and the University of South Carolina at Greenville.

The sample frame is treatment-seeking Veterans with chronic combat-related PTSD at the LBVA or affiliated programs. The sample size of 90 provides adequate power to test the primary hypothesis. Withdrawals prior to beginning intervention will be replaced and withdrawals after beginning intervention will not be replaced.

### Ethical approval, monitoring, and personal information management

This study was approved by the Institutional Review Board and the Research and Development Committee at the Tibor Rubin VA Medical Center. The reporting of this trial is conducted according to Recommendations for Intervention Trials (SPIRIT) guidelines and the SPIRIT-TCM extension (Table 1). Interim study monitoring is by the centralized Data Monitoring Committee (DMC) of VA Office of Research and Development. As noted in the DMC assignment and charter dated 3/24/2016 and 6/23/2016, respectively, the DMC is comprised of 4 members who are selected in part because they have no conflict of interest with the study and who act independently and advise the Director of VA Research about study progress, adverse events, data integrity, and whether to stop and/or start the study. The study project manager will track any adverse and serious adverse events per protocol. Interim analyses for adverse events and study benefits will be conducted at 6-month intervals by study statisticians and, according to charter stopping rules, will inform the study PI if these rules may need to be implemented. No other audits will be conducted. Personal information of subjects will be kept separate in both hard and electronic copies and de-identified in the saved database.

**Table 1:** SPIRIT chart. CAPS-5 is the Clinician-Administered PTSD Scale-5; SCID is the Structured Clinical Interview for Diagnosis; DES-II is the Dissociative Experiences Scale; DRRI is the Deployment Risk and Resilience Inventory-2; CES is the Combat Exposure Scale; LEC is the Life Experiences Checklist; ACE is the Adverse Childhood Experience Questionnaire; MoCA is the Montreal Cognitive Assessment; PCL-5 is the PTSD Checklist-Military-5; PPR is Psychophysiological Response; BDI-II is the Beck Depression Inventory – II; HAM-A is the Hamilton Anxiety Rating Scale; NMCL-SOM is the New Mexico Symptom Checklist – Somatic Scale; The PSQI is the Pittsburg Sleep Quality Index; VR-12 is the Veterans RAND 12-item Health Survey; EDS is the Emotion Dysregulation Scale; McGill is the McGill Pain Questionnaire; Southampton is the Southampton Needle Sensation Questionnaire; AQ is the Aggression Questionnaire; CRS is the Credibility Rating Scale

STUDY PERIOD						
	Enrollment	Allocation and Baseline	Treatment			Follow-up
Timepoint	Week -4 to -1	Week -4 to 0	Week 0	Week 6	Week 12	Week 16
**Screening, Enrollment, Allocation and Baseline Data**						
Screening for Eligibility	X					
Informed Consent	X					
Inclusion and Exclusion						
CAPS-5 [16]	X					
SCID [17]	X					
DES-II [18]	X					
DRRI [19]	X					
CES [20]	X					
Narrative-Exposure, Effects on Health and with Treatment (unpublished)		X			X	
LEC [21]	X					
ACE [22]		X				
MoCA [23]	X					
Allocation		X				
**Interventions**						
Verum Acupuncture						
Sham Acupuncture						
**Assessments: Primary Outcomes**						
CAPS-5 Severity Score [16]		X		X	X	X
PCL-5 [24]		X		X	X	X
**Assessments: Secondary Outcome**						
PPR (startle response)		X		X	X	X
**Assessments: Exploratory**						
BDI – II [25]		X		X	X	X
HAM-A [26]		X		X	X	X
NMCL – SOM [27]		X		X	X	X
PSQI [28]		X		X	X	X
VR-12 [29]		X		X	X	X
EDS [30]		X		X	X	X
McGill [31]		X		X	X	
Southampton [32]			Bi-weekly			
PPR (HR, HRV, SCR)		X		X	X	X
**Assessments: Safety and Control**						
AQ [33].			Weekly		X	X
BDI – II items 9, 11, 17			Weekly		X	X
Satisfaction with Care and Provider Scale (Unpublished)					X	X
Intercurrent Health Resource Use Inventory (unpublished)		X			X	X
**Assessments: Fidelity and Credibility**						
Treatment Fidelity Assessment (Unpublished)		selected				
CRS [34]		selected	session 2	session 24		

CAPS-5 is the Clinician Administered PTSD Scale-5; SCID is the Structured Clinical Interview for Diagnosis; DES-II is the Dissociative Experiences Scale; DRRI is the Deployment Risk and Resilience Inventory-2; CES is the Combat Exposure Scale; LEC is the Life Experiences Checklist; ACE is the Adverse Childhood Experience Questionnaire; MoCA is the Montreal Cognitive Assessment; PCL-5 is the PTSD Checklist-Military-5; PPR is Psychophysiological Response; BDI-II is the Beck Depression Inventory – II; HAM-A is the Hamilton Anxiety Rating Scale; NMCL-SOM is the New Mexico Symptom Checklist – Somatic Scale; The PSQI is the Pittsburg Sleep Quality Index; VR-12 is the Veterans RAND 12-item Health Survey; EDS is the Emotion Dysregulation Scale; McGill is the McGill Pain Questionnaire; Southampton is the Southampton Needle Sensation Questionnaire; AQ is the Aggression Questionnaire; CRS is The Credibility Rating Scale

Chan A-W, Tetzlaff JM, Altman DG, Laupacis A, Gøtzsche PC, Krleža-Jerić K, Hróbjartsson A, Mann H, Dickersin K, Berlin J, Doré C, Parulekar W, Summerskill W, Groves T, Schulz K, Sox H, Rockhold FW, Rennie D, Moher D. SPIRIT 2013 Statement: Defining standard protocol items for clinical trials. Ann Intern Med. 2013;158(3):200-207.

Dai L, Cheng CW, Tian R, Zhong LL, Li YP, Lyu AP, Chan AW, Shang HC, Bian ZX. Standard Protocol Items for Clinical Trials with Traditional Chinese Medicine 2018: Recommendations, Explanation and Elaboration (SPIRIT-TCM Extension 2018). Chin J Integr Med. 2019;25(1):71-79. PMID: 30484022

### Research questions

#### Study objectives

The main objective of this paper is to describe the methodological approach of an RCCT designed to determine the clinical and biological effects of verum acupuncture for combat-related PTSD in US Veterans. Secondary objectives of the RCCT are to determine the effects of acupuncture on symptoms and biology associated with PTSD.

#### RCCT primary hypothesis

The efficacy of verum acupuncture (ACU) for PTSD symptom severity will be large (pre- to post-treatment Cohen’s *d* ≥ 0.8) and significantly better than sham acupuncture (MIN), between-group Cohen’s *d* ≥ 0.30, with 80% probability of detecting a true group difference at p < 0.05 (2-sided).

#### RCCT secondary hypothesis

Compared to MIN, ACU will be associated with a significantly larger change from pre- to post-treatment in peripheral psychophysiological responses (PPRs) (decreased startle by EMG eyeblink during fear conditioning) with 80% probability of detecting a true group difference at p < 0.05 (2-sided).

#### RCCT exploratory hypotheses

1. The efficacy of ACU for symptoms comorbid with PTSD (depression, anxiety, somatic symptoms, sleep and dreaming symptoms, and functional impairment) will be large (pre- to post-treatment Cohen’s *d* ≥ 0.8) and significantly better than MIN (between-group Cohen’s *d* ≥ 0.25).

2. Compared to MIN, ACU will be associated with a significantly larger change from pre- to post-treatment in peripheral psychophysiological responses (PPRs) (decreased heart rate and skin conductance responses and decreased sympathetic activation as measured by skin conductance level and increased parasympathetic activation measured by heart rate variability during baseline and fear conditioning).

### Determining sample size

Extant clinical trials about acupuncture for PTSD—or any condition for that matter—use variable measures and outcomes, making sample size determination challenging. Our own initial study utilized the Posttraumatic Symptom Scale – Self Report (PSS-SR) for The Diagnostic and Statistical Manual of Mental Disorders IV (DSM IV) [35] as the primary outcome, whereas the current study uses the Clinician-Administered PTSD Scale (CAPS-5) for Diagnostic and Statistical Manual of Mental Disorders-5th ed. (DSM-5) [16], metrics of which were not published at the time of study planning. Our first study design was active acupuncture vs. a wait-list control. The current study will use a minimal needling (MIN) sham control to verum acupuncture (ACU), and there are no published data about this comparison in PTSD. A 2010 Cochrane systematic review of over 200 trials investigating 60 clinical conditions found placebos to not have important clinical effects yet may influence patient-reported outcomes in some situations (e.g., pain and nausea). In that review, the pooled relative risk calculated for placebo was 0.93, an effect of only 7% yet significant. Confidence intervals are generally wider in placebo vs. active condition. Several clinical and methodological factors were associated with higher effects of placebo. Since our study includes some of these factors, we reasoned that MIN could provide as much as a 33% reduction in PTSD symptoms with a wider variance than ACU. A conservative prediction would be a mean CAPS-5 reduction of 25 points with ACU and 16.5 points with MIN, which is an 8.5-point between-group post-treatment difference with a pooled SD of 14.4. A less conservative expectation is a 12-point between-group difference and a pooled SD of 15. The conservative assumption will result in pre- to post-treatment Cohen’s *d* = 2.07 within group (ACU) and *d* = 0.59 between group (post-treatment difference), which will result in a rejection of the null hypothesis in favor of the alternative and show a large treatment effect for ACU and a moderate between-group effect size. The conservative assumption requires a total of 90 patients in a two-arm parallel design to provide a probability of 80% to detect a treatment difference at a two-sided 0.05 significance level. The modest assumption requires a total of 50 patients with a power of 0.80 at alpha < 0.05. We will randomize 90 subjects for this trial to be well powered to achieve a more definitive conclusion than previous trials.

### Inclusion criteria

These criteria were meant to recruit a relatively homogeneous yet generalizable sample. Criteria are (1) Veterans age 18 to 55, (2) DSM-5 criteria for chronic PTSD on the CAPS-5, and (3) at least moderate PTSD by having a total CAPS-5 score of ≥ 26 and meeting criteria for each of 4 symptom clusters. Eligible persons will be allowed to have other symptoms that are commonly comorbid with PTSD (e.g., anxiety, mild to moderate depression).

We would have ideally included an older age range of Veterans to increase sample frame and generalizability. However, ANS function (sympathetic/parasympathetic “balance”) begins to change at about age 55 [36–38]. Since acupuncture may work via the ANS, and since secondary and exploratory hypotheses are dependent on ANS functioning, and the ANS age-related changes are difficult to detect in order to rationally exclude subjects, the best choice was to exclude those older than 55 [39].

### Exclusion criteria

These criteria were meant to rule out individuals with characteristics that are known to be PTSD treatment confounds, that might affect biological assessment, that indicate past non-adherence or treatment resistance, or that indicate risk of harm. Criteria are (1) current and past 6-month psychosis; (2) substance dependence within the past 6 months; (3) thyroid disease; (4) decisional incapacity; (5) centrally acting medications that have a potential effect on biological expression (e.g., beta-blockers, opiates, and ≥ 10 mg equivalent of diazepam/day), (6) pain levels requiring opiate medications; (7) known exposure to chemicals or physical trauma that cause neuropsychiatric sequelae; (8) severe depression (Beck Depression Inventory-II score ≥ 30) that is deemed more clinically significant than PTSD; (9) a diagnosed and untreated sleep breathing disorder (SBD); (10) a high risk of a SBD as indicated by snoring ≥ 50% of nights plus one of (a) any witnessed apnea, (b) feeling non-refreshed in the morning ≥ 50 of mornings, or (c) daytime sleepiness indicated by falling asleep with routine tasks such as watching TV or reading; (11) non-response to ≥ 2 evidence-based PTSD treatments (adequate medication of 12 weeks or completion of Prolonged Exposure therapy, Cognitive Processing Therapy, or an intensive program); (12) treatment non-adherence indicated by stopping treatment or > 3 missed appointments in the course of a PTSD treatment; (13) high dissociation as indicated by a score of ≥25 on the Dissociative Experiences Scale – II (DES-II) [18]; (14) past chronic PTSD prior to military service; (15) current *active* psychotherapy for PTSD; (16) having had acupuncture in the past year; or (17) pregnancy. A person who is on a stable dose (6 weeks) of medication for depression, anxiety, PTSD, or sleep and who will continue these medications for the duration of the trial will not be excluded.

### Discontinuation criteria and post-study care

Participation is voluntary, so a subject may discontinue at any time. A subject has 15 weeks to complete the 24 sessions but will not be discontinued for proceeding at a slow pace, as we will evaluate outcomes by the number of sessions completed. Adherence to the protocol is thus tracked as the number of sessions completed. A subject may be withdrawn if they engage in exclusionary criteria (e.g., obtain other PTSD-specific treatment). Subjects may receive ancillary care during the trial if that care is not a specific evidence-based treatment for PTSD (e.g., prolonged exposure therapy). All subjects will be referred back to the initial referral source for post-study clinical care.

### Recruitment and screening

**Fig. 1:**
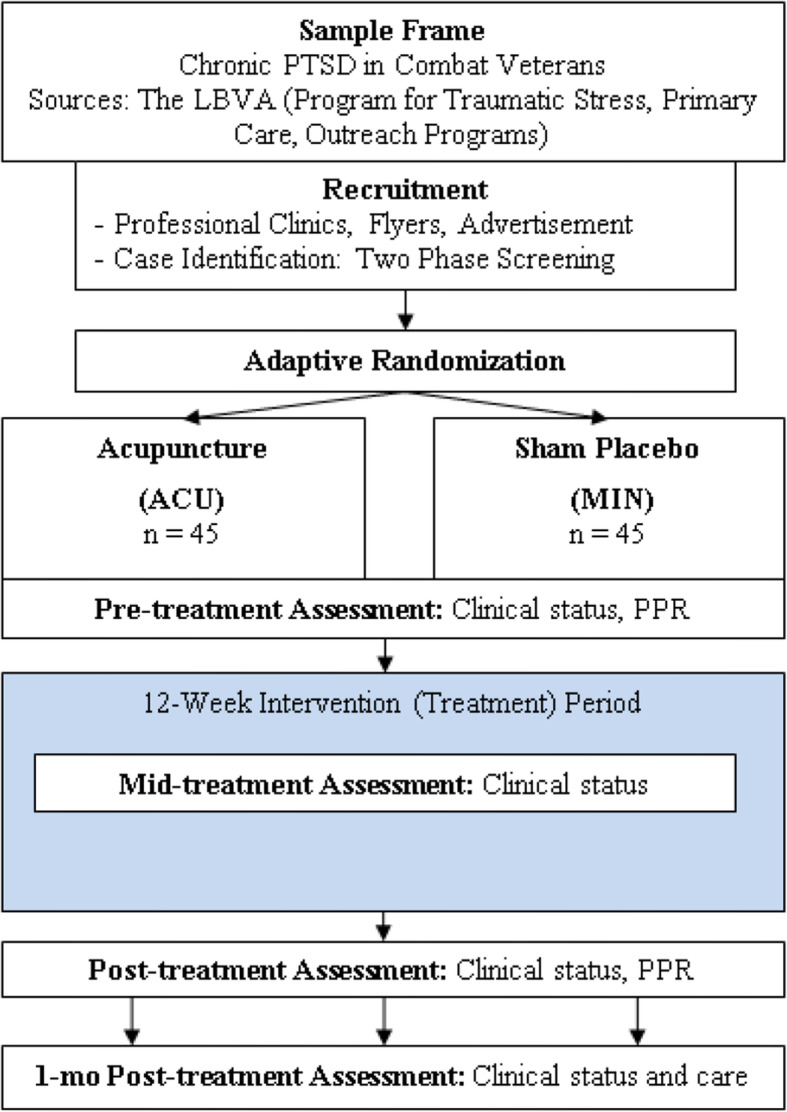
Sample frame and flow of project

Figure 1 shows the flow of subjects through the study. A two-stage detection method will be utilized to identify a sample of PTSD treatment seekers. For stage I, the Program Manager (PM) will respond to interested patients by conducting a phone screen. If a participant meets the initial criteria during the phone screen, the PM will schedule the patient to administer informed consent. Those who consent will be assessed with screening instruments for inclusion and exclusion criteria, availability, and willingness to participate in the study. For stage II, inclusion/exclusion confirmation will be completed by the PM and assessor. The PM will administer the Montreal Cognitive Assessment (MoCA), DES-II, and The Deployment Risk and Resiliency Inventory (DRRI) combat experiences scale and preparedness scale [19] to aid group allocation. The assessor will administer CAPS-5 (past month) and a modified Structured Clinical Interview for Diagnosis DSM-5 (SCID-5) [16]. The PM and/or Primary Investigator (PI) will assess current medications as potential exclusions.

It is not known how many patients will need to be contacted and introduced to the study in order to randomize the desired 90 as subjects [40]. Recruitment will be from sources at the VA proven to be successful in previous studies. Enhancements to recruitment will be to advertise to Veteran groups in the larger Los Angeles area. Retention is best promoted during recruitment by being fully transparent and providing disclosure about procedures, timeline, and study payments. A subject who withdraws will be provided the opportunity to continue providing data on the planned schedule to enhance study data. As part of informed consent, subjects will understand where to go and who to contact for financial and/or clinical compensation if they have been harmed by the clinical trial.

### Randomization and blinding of staff to allocation and data

Subjects will be allocated to the intervention group by a computer-generated adaptive randomization procedure called minimization. Because of the adequate but still modest sample size in this study, simple randomization may not provide group equality on variables that may affect outcome. While minimization can risk subversion or technical error [41], it has been shown to be the best method of ensuring balance between groups for several prognostic factors in small to moderate samples [42, 43], where blocking and stratification are not effective in such trials. With minimization, the treatment allocated to the next participant enrolled in the trial depends on the characteristics of those participants already enrolled to minimize the imbalance across factors. While data about factors that predict outcome in PTSD are not robust, one study in Veterans showed that combat exposure and pre-deployment preparedness accounted for significant outcome variance using standard exposure-based psychotherapy [44]. There are also data suggesting gender effects of emotional disclosure interventions for PTSD in non-Veteran subjects [45]. As such, allocation to the group will use minimization to provide group equality in descending priority on (1) number in each group, (2) combat exposure, (3) pre-deployment preparedness, and (4) gender.

The Data Analyst (DA) will assign a subject the next consecutive study ID number (i.e., SID# 1–90), which will be the only number on hard copies of documents in order to conceal allocation and maintain blind of all investigators and other staff. The DA will create a separate and password-protected database that will contain randomized data on treatment allocation for participants (ACU vs. MIN). One letter is assigned to participants allocated for ACU and another letter is assigned to participants allocated for MIN. The DA will be the only study staff member that will be able to link SID numbers to the assigned treatment arm and the only staff member to know the password for the randomization database. Other study personnel will be blind to this process and will only see the SID#. This procedure will keep the assessor blind to intervention allocation, and investigators and clinicians blind to assessment data to minimize performance and outcome detection bias. Unblinding is possible only if a subject experiences a serious adverse event deemed to be possibly due to the intervention. Unblinding may be conducted by the clinician during or shortly after an ACU or MIN session if the subject has a serious adverse event requiring immediate medical attention, although this is highly unlikely. Unblinding may be conducted by the PI at any time if the subject is experiencing distress from an adverse event that potentially compromises the subjects’ health, safety, or wellbeing. If unblinding occurs, the subject is immediately withdrawn from the intervention though may participate in further data collection.

### Intervention: selection of experimental and control conditions

Selecting a control condition to verum acupuncture (ACU) is not as straightforward as a placebo pill in a medication trial where double blinding is possible. Many factors may be responsible for improvement with acupuncture—and thus could be controlled for—including provider enthusiasm, positive expectation of the patient, extra attention, physiological effects of needling anywhere, physiological effects of needling at the chosen acupoints, and natural history of the illness. The choice of a control group in an acupuncture trial is a critical one and dependent on study aims [46].

Alternative design options we considered include a three-arm design (ACU vs. MIN and a non-needling relaxation group—RELAX), a two-arm design comparing ACU with another type of sham acupuncture (a non-insertive needle), and a comparative effectiveness trial of ACU with a current Evidence-Based Therapy (EBT) for PTSD. However, the three-arm design has potential problems with adequate power and interpreting data if the differences between the ACU and MIN groups are small because we may not have been able to recruit 45 subjects per group at one site. The use of the non-insertive needle is a good option, yet is expensive and labor intensive, and perhaps no better a placebo than MIN. A comparative trial of ACU with a standard EBT is premature; better proof of efficacy for ACU for PTSD needs to be first established. There are other important questions and research designs about the effects of ACU, such as evaluating the importance of meridians versus individual points, the validity of point fatigue, the mechanisms of action in specific biological systems, or the most effective dose. These should be addressed after efficacy is established.

Sham procedures are not fully inactive [47]. There are essentially three ways to conduct sham acupuncture using needles. Two involve needle insertion: (1) insertion and manipulation in the same manner but at purportedly “irrelevant” points, usually a few millimeters from actual verum points, or (2) superficial insertion at verum points, usually without manipulation in order to not elicit the “DeQi” sensation. Each, or a combination of each, has been labeled “minimal needling.” Investigators have also used various gauge-size needles and very small monofilaments as insertive shams. The third type of sham (3) is non-insertive, where a blunt needle within an adhesive O-ring mechanism hits the skin and retracts without puncture. When this sham is used, the needles for verum acupuncture are also administered via the adhesive O-ring for blinding. Both insertive and non-insertive procedures probably influence expectation, sensation, and contextualization, and both have shown effects in brain areas controlling sensation, cognition, and affect [48]. While non-insertive needles have been developed and shown to be valid shams from the subjective patient view (e.g., the “Streitberger” and the “Park Sham Device”) [47, 49], these are far more cumbersome and very expensive compared to minimal needling. And, research has shown clinical differences between verum ACU and MIN needling techniques, for example in studies of chemotherapy-induced emesis control [50] and blood pressure control [51]. In the Shen study where all patients were provided pharmacotherapy for emesis, emesis events and emesis-free days were significantly better with ACU vs. MIN vs. pharmacotherapy only (events 5 vs. 10 vs. 15, respectively; free days 55% vs. 29% vs. 20%, respectively). MIN is the best available sham control to verum ACU because they both are similar in all aspects except that MIN includes irrelevant acupoints and shallow insertion without trying to obtain DeQi.

Acupuncture needles can be stimulated manually by rotating, lifting, and/or thrusting or by using an electrical stimulator or both. The majority of recent acupuncture studies in animals and humans use electrical stimulation, which delivers more consistent stimuli to afferent nerves than the manual-only approach and may have a stronger impact on inhibiting neural cell activities than the manual approach (MA) [52].

### Intervention: protocols

#### Experimental group: verum acupuncture (ACU): Table 2

Individual treatment sessions are 1 h twice per week for 12 weeks and reflect clinical practice with an interview (10 min), pulse and tongue observation (5 min), standard needling, and needle retention (30 min). Subjects receive a standard acupuncture point prescription defined in our previous study and chosen for the most likely TCM diagnostic patterns for PTSD. An alternating by session front and back treatment will be used to avoid point fatigue (tolerance due to frequent use). Bringing DeQi for each point is desired. The front treatment is comprised of 11 needles, bilaterally at LV3, PC6, HT7, ST36, SP6, and at the single Yintang point; the back treatment is 14 needles, bilaterally at GB20 and UB14, 15, 18, 20, 21, and 23. In addition to the standard points, three additional points (chosen from a list of 15 points) will be chosen to address a subject’s constitution based on the TCM diagnostic patterns, available in original published reports [7, 8].

**Table 2:** Acupuncture points for the treatment of post-traumatic stress disorder

**Primary patterns (standard points for all subjects)**						
	**HT Shen disturbance**	**LV Qi stagnation**		**Kidney deficiency**	**Grounding points/Qi and blood deficiency**	
**Front points**	HT7, PC6 and Yintang (even)	LV3, (PC6) (even)			ST36, SP6 (even)	
**Back points**	UB14, 15 (even)	GB 20, UB18 (even)		UB 23 (reinforce)	UB 20, 21 (even)	
**Secondary patterns (up to three points chosen)**						
	**LV overacting on SP**	**LV overacting on ST**	**ST fire**	**LV fire**	**Phlegm-heat**	**Phlegm-damp**
**Front points**	LV13 (reinforce)	LV14 (reduce)	ST44 (reduce)	LV2 (reduce)	ST40 (reduce)	SP9 (reduce)
**Back points**	UB18 (reduce) UB20 (reinforce)	UB18 (reduce) UB21(reduce)	Du 14 (reduce) UB21 (reduce)	Du 14 (reduce) UB18 (reduce)	Du 14 (reduce) UB21 (reduce)	UB20 (reduce)
	**HT Yin/blood deficiency**	**SP Qi/Yang deficiency**	**KI Yin/essence deficiency**	**KI Yang/Qi deficiency**	**LV Yin/blood deficiency**	**ST Yin deficiency**
**Front points**	HT6 (reinforce)	SP3 (reinforce)	KI6 (reinforce)	KI7 (reinforce)	LV8 (reinforce)	ST44 (reinforce)
**Back points**	UB17 (reinforce) UB15 (reinforce)	UB20 (reinforce) UB23 (reinforce)	UB52 (reinforce) UB23 (reinforce)	Du4 (reinforce) UB23 (reinforce)	UB17 (reinforce) UB18 (reinforce)	UB21 (reinforce)

#### Control group: sham minimal needling (MIN): Table 3

Individual treatment sessions are 1 h twice per week for 12 weeks. Three elements define minimal needling in this study. The first is the location of the needles, which are 2 cm lateral or medial to actual reference points, which are not associated with PTSD and are not expected to affect PTSD symptoms. The second is the superficial insertion depth (< 0.25 inch) compared to ACU. The third is the relative absence of stimulation due to the depth and the use of a non-functioning stimulator to complete the sham effect. The actual distance between the acupuncture reference point and the sham site needled is approximate, with consideration given to (1) nearby acupuncture meridians; (2) superficial or deep anatomical features such as skin abrasion, visible vessels, or palpable nerves; and (3) the proportions of each patient’s body. For example, the lung and large intestine meridians are close to one another at the location of LU 7, so care is taken to choose a sham needling site between the two meridians. Some discretion is afforded the acupuncturists with regard to sham needling sites near SI 4 and SJ 12 based on the patient’s selection of body position on the table, and in this case, we have listed the sham site as either anterior or posterior, with care taken to remain off-meridian. The acupuncturist will perform no manipulation to obtain DeQi and sham needles will only be adjusted to more superficial depth to minimize reported sensations such as stinging or irritation. The protocol also uses 11 front and 14 back points to match the number and body position (alternating prone and supine) of points in the ACU group.

**Table 3:** Sham (MIN) points for the treatment of post-traumatic stress disorder

**Point locations-front treatment**									
**Reference point**	SI19 (left)	SJ3	SI4	LU7	LU6	ST25	ST34	SP7	
**Adjusted location**	1 cm anterior	1 cm lateral	1–2 cm distal and 1 cm posterior *or* anterior*	1 cm proximal and 1 cm posterior	1 cm lateral	2 cm lateral	2 cm medial	1–2 cm posterior	
**Point locations-back treatment**									
**Reference point**	SI15	SI11	SI9	SJ12	GB30	GB32	GB33	GB34	UB57 (left)
**Adjusted location**	1–2 cm lateral	1–2 cm lateral	1–2 cm lateral	1–2 cm anterior *or* posterior*	2 cm lateral	2 cm posterior	1–2 cm posterior	2 cm posterior	2 cm lateral

#### Informing subjects and maintaining blind about group allocation

Blinding may be compromised unless care is taken during allocation, informed consent, and the actual sessions. We will adopt practical steps after McManus et al. [53], which include how to set up the room and materials prior to treatment, the materials to have on hand, and how to insert, manipulate, and remove the needles. These procedures were shown in an RCT to be successful by having 71% of those receiving sham and 81% of those receiving verum (p = 0.20) believe they received “real” treatment. Another important procedure for maintaining subject blinding is the construction and delivery of the informed consent. Subjects will all be informed that there are “two types of treatments that involve acupuncture needles” or “two forms of treatment with needles” that will be used during this clinical trial, and that they will be randomly allocated to receive either one or the other treatment. Subjects will not be offered the name or type of the interventions. As part of the informed consenting process, participants will be told researchers do not know which group will be more beneficial than the other and that figuring this out is the goal of the study. Any questions they ask about the needles or technique will be responded to with a structured answer that has been part of the training of all staff prior to the trial, such as “as mentioned in the consent, there are two types of needle methods being used, and you may receive either one during any given session.”

#### Controlling for non-specific treatment-related effects

It is critical for those in the MIN group to receive an equivalent amount and kind of time, empathy, setting, and assessment as those in the verum ACU group. Both ACU and MIN protocols will include language currently in our acupuncture clinical trials manual for greeting, interacting with, and closing a session.

### Outcome measures and data entry, integrity, and analyses

#### Primary outcome: clinical

The CAPS-5 [16] is a structured diagnostic interview assessing DSM-5 criteria for PTSD. CAPS-5 items are rated with a single severity score, in contrast to previous versions of the CAPS which required separate frequency and intensity scores for each item that were either summed to create a symptom severity score or combined in various scoring rules to create a dichotomous (present/absent) score. CAPS-5 has 20 symptom items, each rated from 0 (absent) to 4 (severe). A rating of > 2 is considered a positive score for diagnostic purposes. There are 4 symptom clusters: the Criterion B (re-experiencing) severity score is the sum of individual scores for items 1–5, the Criterion C (avoidance) severity score is the sum of items 6 and 7, the Criterion D (negative alterations in cognitions and mood) severity score is the sum of items 8–14, and the Criterion E (hyperarousal) severity score is the sum of items 15–20. The DSM-5 diagnostic rule requires the presence of at least one Criterion B symptom, one Criterion C symptom, two Criterion D symptoms, and two Criterion E symptoms in addition to other impairment criteria.

#### Secondary outcome: biological, psychophysiological response (PPR) startle eyeblink

Acoustic startle responses will be measured as part of an established fear conditioning paradigm that employs visual conditioned stimuli (CSs) paired with a 250-ms airblast with an intensity of 140 psi directed to the larynx (unconditioned stimulus; US), a protocol that has been used with numerous populations both psychiatrically healthy and trauma-exposed [54]. This paradigm has been shown to be sensitive to treatment effects for PTSD [55]. Startle responses are obtained from EMG recordings during eyeblink muscle contractions induced by a 108-dB burst of white noise [54] that is present on each trial of the fear conditioning task. PPR data will be collected, amplified, and digitized by the Biopac MP150 system for windows (Biopac Systems, Inc., Goleta, CA), using the EMG, electrodermal activity (EDA), and electrocardiogram (ECG) modules. Data will be exported to Mindware software (Mindware Technologies LTD, Gahanna, OH) for data reduction and generation of analyzable variables. The final output will be analyzed to assess fear-potentiated startle (the relative increase in the startle magnitude elicited in the presence of conditioned stimuli) as well as the reduction of fear during extinction.

EMG startle eyeblink responses will be recorded using two 5-mm silver/silver chloride (Ag/AgCl) electrodes placed over the orbicularis oculi muscle of the right eye. One electrode will be placed directly below the pupil in forward gaze while the other will be placed about 1 cm lateral to the first. Both electrodes will be placed as close to the eye as possible while still allowing the participant to close his or her eyes comfortably. Skin conductance will be acquired through two Ag/AgCl electrodes placed on the palm, and ECG will be collected in the lead 2 position, with active electrodes placed below the collarbone and on the torso. Impedance between the two EMG electrodes will be measured and deemed acceptable if below 10 kΩ. EMG data will be filtered between 28 and 500 Hz and peak amplitude occurring between 20 and 200 ms after startle probe onset will be scored.

#### Exploratory outcomes

We will evaluate the efficacy of ACU for symptoms comorbid with PTSD (depression, anxiety, somatic symptoms, sleep and dreaming symptoms, and functional impairment) and for other PPR (heart rate and skin conductance responses to assess sympathetic activation and parasympathetic activity as measured by high-frequency heart rate variability (HF-HRV) during baseline and fear conditioning).

#### Treatment fidelity and subject expectancy

Assuring treatment fidelity is important in clinical trials. With one practitioner, accuracy and consistency are required, but of course there is no need for inter-therapist reliability. Study acupuncturists are well-trained, research experienced, and will be further trained on study protocol by the investigators. At least five volunteer patients each for ACU and MIN will be treated and observed by each therapist; landmarks, needle insertion and manipulation, electrical stimulation, and needle removal will be judged and discussed for each case. Second, non-specific elements of the intervention, such as instructions for treatment preparation, and interactions during treatment will be reliably delivered by using clinical scripts adapted from our first study. Third, all treatment sessions will be videotaped. The first 5 and a random selection of 15% (18 total cases) thereafter chosen by a computer randomizer will be scored by a co-investigator (AH) using the Fidelity Assessment Rating Form adapted for this study.

Treatment credibility [34] will be examined as has been done in previous acupuncture studies before session 1 and after sessions 2 and 24 to explore the effects of expectancy by group and total sample on the outcome.

#### Data collection, entry, and integrity

Data will be collected by program managers and assessors who are trained from other funded studies. Data will be entered into a password-protected customized spreadsheet by a research assistant using the data code book developed by the study team and statisticians. Another assistant will conduct a second independent pass of data checking. Discrepancies will be corrected by both assistants reviewing the source documents together. Once corrected, the spreadsheet is saved as final. A copy is made for all subsequent entries. The spreadsheet is formatted for seamless transfer to the statistical database used by study statisticians, who will check the copy before conducting analyses. Source data forms and data management protocols are kept in both electronic and hard copy form in the research offices for review in the case of an audit or interest by other investigators.

#### Data analyses

General linear mixed models (GLMM) are capable of handling multiple underlying distribution and model structures through link functions, such as repeated measures random effects models of continuous outcomes (identity link), repeated measures logistic models (logit link), and Poisson and negative binomial models (log link). In addition to modeling global fixed effects across subjects, GLMM can also model individual subject random effects. Cox proportional hazards modeling can be used to evaluate the effect size of the treatment by allowing subjects to contribute their information to the model while they are being observed and censored once they are no longer being observed (loss to follow-up or end of the study period). In the case where dropouts may be associated with the treatment assignments, we will leverage intention to treat methodology and construct a piecewise random effects model with both “on-” and “off-treatment” slopes. GLMM will be used to evaluate the primary clinical hypothesized effects of treatment (ACU) on the clinical outcome of PTSD symptom severity (CAPS) over time (mid- and end-treatment, and 1-month follow-up), controlling for baseline severity of symptoms and demographic characteristics (e.g., age, gender) in comparison with the placebo control group (MIN) with the assumption of intent-to-treat. Cohen’s d within and between subjects will be calculated. Interaction terms will be included in the models to evaluate treatment fidelity and treatment expectancy as potential moderators. GLMM will also be used to evaluate the secondary biological hypothesized effects of treatment on pre- to post-treatment in PPR (decreased EMG eyeblink). These same statistical procedures (GLMM, survival analysis, and Cohen’s d with intent to treat) will be applied to evaluate exploratory outcomes: clinical symptoms comorbid with PSTD, PPR (HR, HRV, SCR), and PTSD diagnosis. While this is a comprehensive initial statistical plan for primary, secondary, and exploratory outcomes, investigators may contact the PI for further details if desired and will be able to further view statistical analytic plans in the subsequent papers.

#### Possible protocol changes and communication

Any necessary changes to eligibility criteria, outcomes, data collection, and/or management or data analyses will be reported to the Institutional Review Board (IRB) at the Long Beach VA and to the centralized DMC where appropriate.

## Discussion

This will be the first single-blinded RCCT study of acupuncture for PTSD in a Veteran population using a sham acupuncture control, a standardized protocol, and assessing both clinical (CAPS-5) and biological outcomes, primarily fear-potentiated startle and the reduction of fear during extinction. Of the eight known published clinical trials of acupuncture for PTSD, three recruited Veterans and the dose of acupuncture was highly variable. Two used a sham comparator. Prisco and colleagues used group auricular acupuncture vs. sham and wait-list control on sleep quality and medication use in Veterans from the Iraq and Afghanistan conflicts, not primarily for PTSD [56]. Huang and colleagues reported the benefit of acupuncture vs. a sham control in an RCCT, but this was also primarily for persistent disturbed sleep in Veterans with mild traumatic brain injury [57]. None of the extant studies evaluated both clinical and biological outcomes.

A stated limitation of acupuncture trials is the inability to use a double-blind design. A core component of effective acupuncture is obtaining the sensation of DeQi [58], which cannot be achieved by a blind method with current knowledge and techniques. This is similar to psychotherapy research where the therapist is trained to elicit specific responses from the subject and cannot be blinded to treatment. A study could have multiple arms with each therapist working only in one arm and blinded to what arms exist, yet this would risk other kinds of bias.

The current study will thus address previously noted limitations in early studies by utilizing a placebo (sham) arm, gold-standard clinical assessment as the primary outcome, and a biological outcome. In addition, the current study will have at least two acupuncturists, allowing for comparison, and will monitor and assess protocol adherence. Though limitations may persist, this study presents the most robust methodological integration of best practices available in RCTs of PTSD therapies to mitigate bias and achieve confidence in effect sizes. Methodological repetition will be necessary to corroborate and enhance the understanding of these methods and forthcoming findings in future studies.

## Trial status

This is protocol version 1.0, 5 June 2020. The initial release was on 17 August 2016. Recruitment started on 5 April 2018, with an anticipated primary completion date of 30 September 2021.

## Supplementary Information

**Additional file 1.** Ethical Approval Document 1 of 2. IRB Approval Letter.

**Additional file 2.** Ethical Approval Document 2 of 2. Data Monitoring Review Approval Letter.

**Additional file 3.** Original Funding Documentation. Merit Review Award Letter.

**Additional file 4.** Original Funding/Monitoring Documentation. DMC Charter.

## Data Availability

Only the core team will have access to the final dataset. Data and materials may be made available from the corresponding author upon request and with VA IRB approval only. Data sharing within VA for full trial or individual participant will conform to VA ORD guidelines.

## Abbreviations

PTSD: Post-traumatic stress disorder
EMG: Electromyographic
RCCT: Randomized controlled clinical trial
TCM: Traditional Chinese medicine
CBT: Cognitive behavior therapy
HPA: Hypothalamic-pituitary-adrenal
ANS: Autonomic nervous system
CNS: Central nervous system
LBVA: Long Beach VA Healthcare System
ACU: Verum acupuncture
MIN: Sham acupuncture
PPR: Psychophysiological response
PSS-SR: Posttraumatic Symptom Scale – Self Report
DSM IV: The Diagnostic and Statistical Manual of Mental Disorders IV
CAPS-5: Clinician-Administered PTSD Scale
DSM V: The Diagnostic and Statistical Manual of Mental Disorders
SBD: Sleep breathing disorder
DES – II: Dissociative Experiences Scale – II
PM: Program Manager
MoCA: Montreal Cognitive Assessment
DRRI: Deployment Risk and Resiliency Inventory
SCID-5: Structured Clinical Interview for Diagnosis – 5
PI: Primary Investigator
DA: Data Analyst
EBT: Evidence-Based Therapy
MA: Manual approach
CSs: Conditioned stimuli
US: Unconditioned stimulus
EDA: Electrodermal activity
ECG: Electrocardiogram
AG/AgCl: Silver/silver chloride
ms: Millisecond
HF-HRV: High-frequency heart rate variability
