# Supplementary material for: Acupuncture for combat post-traumatic stress disorder: trial development and methodological approach for a randomized controlled clinical trial
Source: Trials. 2021 Sep 6;22:594. doi: 10.1186/s13063-021-05394-3 (PMC8419889; doi:10.1186/s13063-021-05394-3)
Supplement: Supplementary file 1 — Additional file 1. Ethical Approval Document 1 of 2. IRB Approval Letter. [file 13063_2021_5394_MOESM1_ESM.pdf]

IRB (Subcommittee on Human Studies)
Long Beach VAMC Research Health Care Group (151)
5901 East Seventh Street • Long Beach, CA 90822 • 562-826-5801 • Fax: 562-082-6567

IRB APPROVAL - Previously Tabled Protocol (Initial Review)

Date: June 21, 2016
From: Kathleen Herron, M.D., Member
Investigator: Michael Hollifield, M.D.
Protocol: Acupuncture for PTSD in Combat Veterans
ID: 01414 Prom#: N/A Protocol#: N/A

The following items were reviewed and approved at the 06/09/2016 meeting:

- RDIS - Intial Review (04/04/2016)
- Abstract - IRB Stipulations (05/31/2016)
- Prospectus - Pre-Reviwer Changes (05/03/2016)
- Application for IRB Review - IRB Stipulations (05/31/2016)
- Impact Estimation Worksheets - Pathology-Intial Review (04/06/2016)
- Impact Estimation Worksheets - Pharamacy-Intial Review (04/06/2016)
- Impact Estimation Worksheets - Radiology-Intial Review (04/06/2016)
- Consent Form - IRB Stipulations (06/21/2016)
- HIPAA Authorization - IRB Stipulations (05/31/2016)
- HIPAA Revocation - Intial Review (04/04/2016)
- Narrative - Intial Review (04/04/2016)
- Advertisement - Pull Off Contact Info Flyer-Intial (04/04/2016)
- Advertisement - Intial Review-Volunterrs Needed for Research Flyer (04/04/2016)
- Questionnaire / Survey - Aggression Questionnaire-Intial Review (04/04/2016)
- Questionnaire / Survey - BDI-II- Intial Review (04/04/2016)
- Questionnaire / Survey - Clinician-Administered PTSD Scale for DSM-5-Intial (04/04/2016)
- Questionnaire / Survey - Hopkins Symptoms Checklist 25-Intial Review (04/04/2016)
- Questionnaire / Survey - New Mexico Symptoms Checklist-Intial Review (04/04/2016)
- Questionnaire / Survey - Pittsburgh Sleep Quality Index-Intial Review (04/04/2016)
- Questionnaire / Survey - Veterans Rand 12-Item Health Survey(VR-12) (04/04/2016)
- IRB Stipulations- Memorandum for Projects Off-Site (05/31/2016)
- IRB Stipulations-Memo conducting work offsite (05/31/2016)
- IRB Stipulations-RDIT (05/31/2016)
- Acupuncture for PTSD in Combat Veterans-Intial (04/04/2016)
- Investigators Device Plan-Intial Review (04/04/2016)
- Inclusion/Exclusion Criteria - Intial Review (04/04/2016)

Conditions of Approval are attached. These conditions are further detailed in the HHS, FDA, and VA regulations, which are available in the Research Office.

The VA Long Beach Healthcare System IRB is not connected with, has no authority over, and is not responsible for human research conducted at any other institution, except where a Memorandum of Understanding specifies otherwise. Separate consent forms, initial reviews, continuing reviews, amendments, and reporting of serious adverse events are required if the same study is conducted at multiple institutions.

Approval is granted for a period of 12 months and will expire on 06/08/2017. Your Continuing Review is scheduled for 04/13/2017.

The protocol was determined to have the following level of risk:
Moderate

The following other committee reviews are scheduled:
Research & Development Committee [07/07/2016]

Approval by each of the following is required prior to study initiation:
IRB (Subcommittee on Human Studies)
Subcommittee on Research Safety [Approval Granted 04/13/2016]
Research & Development Committee

Approval for study initiation is contingent upon your compliance with the requirements of the Research Service for the conduct of studies involving human subjects.

Kathleen A Herron

Kathleen Herron, M.D., Member

The VA Long Beach Healthcare System IRB is not connected with, has no authority over, and is not responsible for human research conducted at any other institution, except where a Memorandum of Understanding specifies otherwise. Separate consent forms, initial reviews, continuing reviews, amendments, and reporting of serious adverse events are required if the same study is conducted at multiple institutions.
