# Supplementary material for: Acupuncture for combat post-traumatic stress disorder: trial development and methodological approach for a randomized controlled clinical trial
Source: Trials. 2021 Sep 6;22:594. doi: 10.1186/s13063-021-05394-3 (PMC8419889; doi:10.1186/s13063-021-05394-3)
Supplement: Supplementary file 2 — Additional file 2. Ethical Approval Document 2 of 2. Data Monitoring Review Approval Letter. [file 13063_2021_5394_MOESM2_ESM.pdf]

Department of Veterans Affairs

MEMORANDUM

Date: July 7, 2016

From: CSR&D Centralized Data Monitoring Committees

Subj: DMC Charter for Merit Review Study

To: **Michael Hollifield, M.D.**

A PBN Data Monitoring Committee (DMC) charter has been written for your study and approved by the DMC. This charter explains how the DMC will operate for your study and also describes specific reporting requirements you have in order for the DMC to monitor your study properly. Please find attached the charter for your study **"Acupuncture for PTSD in Combat Veterans"** and take the time to review it.

We request that you sign the document electronically, or print, sign and re-scan it so that it can be returned to the DMC Office no later than **Thursday, July 14, 2016**. You should also keep a copy for your records.

Please note that any changes to the protocol must be communicated to the DMC for approval; the changes should be summarized on the Protocol Change Form (attached). The approved changes will become part of the charter documentation.

If you have any questions, please feel free to contact me at the email address below.

Sincerely,

Yvonne Lucero, M.D.
Centralized DMC Administrator
Hines CSPCC
yvonne.lucero@va.gov
Ph: (708) 202-4897
