# Supplementary material for: Acupuncture for combat post-traumatic stress disorder: trial development and methodological approach for a randomized controlled clinical trial
Source: Trials. 2021 Sep 6;22:594. doi: 10.1186/s13063-021-05394-3 (PMC8419889; doi:10.1186/s13063-021-05394-3)
Supplement: Supplementary file 3 — Additional file 3. Original Funding Documentation. Merit Review Award Letter. [file 13063_2021_5394_MOESM3_ESM.pdf]

Department of Veterans Affairs

Memorandum

Date: MAR 10 2016

From: Acting Director, Clinical Sciences Research and Development (CSR&D) (10P9C)

Subj: Assignment to CSR&D Data Monitoring Committee

To: ACOS/Research, VA Medical Center, Long Beach, CA (600/151)

1. You have been notified that Dr. Hollifield's Merit Review Award project entitled "Acupuncture for PTSD in Combat Veterans" has been approved for funding. This project has been assigned to the CSR&D Data Monitoring Committee (DMC) for the entire duration of the study. We are especially interested in working with you to ensure proper implementation and oversight of this project.

2. Please note that that CSR&D as the study sponsor is responsible for overall administrative and scientific oversight of this project. The CSR&D DMC will monitor this study for safety, efficacy and progress. The DMC is coordinated by Dr. Domenic Reda and his staff at the Hines VA Medical Center. Dr. Reda will contact your research office with necessary details concerning the DMC.

3. This study's assignment to the DMC does not affect the project budget in any way. This is a service that is provided by CSR&D to ensure independent oversight of the safety and integrity of this project. The DMC will make recommendations regarding this study to me, although local Institutional Review Board (IRB) oversight will continue.

4. Our office is interested in ensuring the success of our funded scientific projects. Progress reports must be furnished on an established basis with our office and the DMC. A final outcome paper for this trial must also be received when available.

5. We look forward to the results of this interesting study. Please direct questions and or comments to Theresa Gleason, Ph.D. at (202) 443-5697 or by e-mail at theresa.gleason@va.gov.

Theresa Gleason, Ph.D.
Acting Director, Clinical Sciences Research and Development Service

Cc: Domenic Reda, Ph.D.
Michael Hollifield, M.D.
