# Supplementary material for: Acupuncture for combat post-traumatic stress disorder: trial development and methodological approach for a randomized controlled clinical trial
Source: Trials. 2021 Sep 6;22:594. doi: 10.1186/s13063-021-05394-3 (PMC8419889; doi:10.1186/s13063-021-05394-3)
Supplement: Supplementary file 4 — Additional file 4. Original Funding/Monitoring Documentation. DMC Charter. [file 13063_2021_5394_MOESM4_ESM.pdf]

DEPARTMENT OF VETERANS AFFAIRS

Clinical Sciences Research & Development (CSR&D)

Data Monitoring Committee Charter

Version 1.0

Date: 6/23/16

Official Charter for the Study entitled

"Acupuncture for PTSD in Combat Veterans"

Principal Investigator Name:

Michael Hollifield, M.D.

DMC Charter for "Acupuncture for PTSD in Combat Veterans"

1. Introduction

This document constitutes the Clinical Sciences Research & Development (CSR&D) Data Monitoring Committee (DMC) Charter for the study "Acupuncture for PTSD in Combat Veterans." Currently this study is approved to be conducted at the Long Beach Healthcare System; the complete study is expected to take 5 years. However, it is recognized that the study location(s) and planned end date may change as the study progresses.

This Charter may be reviewed periodically and updated when there are any changes in DMC or study procedures for the duration of the study. All version updates to this document are tracked in the table below.

All information contained below is specific to this study, thus will be referred to as "the study" within this document.

Version Number	Date Need for Change was Identified	Details of Change	Date Change was Finalized
1.0		Draft Initial Charter	

2. Purpose and Responsibilities of the DMC

The DMC identified in this Charter for the study "Acupuncture for PTSD in Combat Veterans" is primarily responsible for safeguarding the interests of study participants, assessing the safety and efficacy of trial interventions, monitoring the progress of the study. This DMC will conduct reviews of this clinical trial and recruitment will be monitored every 4 months. This DMC will serve as an independent advisory group to the Acting Director, CSR&D, and is required to provide recommendations about starting, continuing, and stopping the study. The DMC will:

- Review the study protocol, pertinent accessory documents, and plans for data and safety monitoring
- Review methodology used to help maintain the confidentiality of the study data and the results of monitoring by reviewing procedures put in place by investigators to ensure the privacy of study participants

- Monitor study design, procedures and events to maximize study safety and minimize risks to study participants

- Evaluate the progress of the study, including periodic assessments of data quality and timeliness, participant recruitment and retention, participant risk versus benefit, performance of the study site(s), and other factors that may affect study outcome

- Consider factors external to the study when relevant information becomes available, such as scientific or therapeutic developments that may have an impact on the safety of the participants or the ethics of the study

- Review serious adverse event documentation and safety reports, and make recommendations regarding protection of the safety of the study participants

- Report to the Acting Director, CSR&D, and the Principal Investigator (PI) on issues concerning study safety progress

- Evaluate and report to the Acting Director, CSR&D and the study PI on any perceived problems with study conduct, enrollment, sample size and/or data collection

- Provide to the Acting Director, CSR&D and the PI a recommendation regarding continuation conditionally or unconditionally, probation, termination or other modifications of the study based on the cumulative experience including the observed beneficial or adverse effects of the treatment under study; as well as data observations that indicate the likelihood of definitively addressing the goals of the study

This DMC is responsible for identifying mechanisms for the completion of various tasks that will impact the safety and efficacy of study procedures and overall conduct of the study.

3. CSR&D DMC Organization

The DMC for this study is composed of the members listed in Table 1 (see Appendix). Table 1 lists the role and responsibility of each DMC member. Refer to Figure 1 in the Appendix for the organizational relationship between this CSR&D DMC and study stakeholders.

Committee membership for this DMC is composed of a Chairperson, voting, and advisory or non-voting members. Voting members and advisory members for this DMC may attend closed sessions. Voting and advisory members will have access to unblinded data, upon DMC request, during closed sessions. The Administrator for this Data Monitoring Committee and the respective DMC Manager will provide an unbiased staff interface for DMC members and the study team, especially during executive committee sessions. The DMC Managers will work with CSR&D Program Staff who prepare the meeting minutes to ensure timely transmission of final committee recommendations to the Acting Director, CSR&D for approval.

4. Communications

Communications between this CSR&D DMC and the PI for the study will be conducted through the DMC Administrator and DMC Managers. All documentation needed for scheduled meetings from the PI will be sent to the DMC Manager no later than 30 days prior to the scheduled meeting day. The DMC Manager will distribute requisite documentation to all reviewers for all scheduled DMC meetings. The study PI and study team will not communicate directly with DMC members about the study, except when making presentations or responding to questions at open sessions of the DMC. DMC scheduled meetings or sessions may occur in person or via teleconference. (See Section 7 for a definition of Open versus Closed Sessions of the DMC). The study biostatistician may, however, attend closed sessions when data must be unmasked for committee voting purposes. The PI will communicate with the DMC through the DMC Administrator or Manager.

The DMC **does not approve** modifications to a study but **does review** modifications at convened meetings. **Administrative requests for certain time and budget dependent approvals, such as no-cost extensions and at-cost extensions, must be submitted by the PI directly to Central Office and approved by the Acting Director, CSR&D.** CSR&D, as the sponsor, has the final authority to approve such PI requests. Modification requests should also be submitted simultaneously to the DMC Office, as the DMC membership is to be informed of any plans for substantive change prior to the change. The DMC reviews major statistical and monitoring modifications to the study plan including changes to the sample size, primary analysis, and/or interim monitoring and makes recommendations accordingly.

5. Conflict of Interest (COI)

Any real or apparent COI's of DMC members with the study are identified at the onset of review of the study. Prior to each meeting, all members of the "Acupuncture for PTSD in Combat Veterans" DMC will have an opportunity to state whether they have developed any new COI's since the previous meeting. As a new COI is identified, it must be documented and provided to the DMC Manager for the study. If a new conflict is reported, the Chairperson and/or DMC Office Staff will determine if the conflict limits the ability of the DMC member to participate in the discussion of the study prior to any committee actions.

6. Scheduling, Quorum, and Organization of Meetings

The purpose of the first meeting for the DMC for this study is to:

- Amend, review, and discuss and sign the Charter, although this may not be accomplished until after the DMC has made its initial recommendations for a study

- Provide an overview of study activities as well as discuss details of the study's safety plan (see also Safety Plan, under Appendix, for specifics)

- Review and make recommendations about the study protocol(s)

- Determine the type and frequency of study data summaries that must be provided to the DMC for its reviews

- Determine the frequency of formal interim analyses, if applicable, and whether data will or will not be masked to reveal the identity of randomized groups.

In addition to familiarizing committee members with the study and study team at the first meeting, the DMC will determine logistics for following meetings (both in-person and teleconferences), which will be documented in Table 2 (See Appendix).
It is expected that all DMC members who are identified in Table 2 will attend every committee meeting. However, it is recognized that this may not always be possible. Quorum must be reached in order for an item to be voted on. In order for quorum to be present, greater than 50 percent of committee voting membership is required. This quorum, of over 50 percent of committee members, must include two clinicians and one biostatistician for all voting purposes.

7. Materials and Protocol for CSR&D DMC Meetings

The agenda for CSR&D DMC meetings and calls will be drafted by the DMC Office in consultation with the PI and study team.

Recurring Data Reviews:

The PI and/or study team will furnish the DMC Office with the following reports, at a minimum of 30 days, before each meeting or conference call in order to allow members adequate time to review and prepare for the meeting at hand. Meeting materials will include the following reports and data:

- Adverse Events (AE) Data

- Serious Adverse Events (SAE) Data

- Unexpected Problems (UP) Data

- Graphed Enrollment Data (Actual versus Projected)

- Recruitment and Retention Data

- Statistical Analysis of Study Progression

The DMC will review some or all of the above information at meetings depending on the type of review being conducted to ensure proper conduct of the study. The DMC may also apply other metrics in making its assessments, such as targeted enrollment versus actual enrollment.

Meeting Procedures:

DMC meetings will be organized into "open" and "closed" sessions. Definitions for each meeting type are included below. The meeting type will be identified by the DMC Manager when providing the DMC Chairperson and members with the meeting agenda.

Open Sessions: These sessions of the committee are open to the PI and the study team. Specific data summaries which will always be outlined at the beginning of the session will be reviewed. These data summaries will be reported in aggregate format and not by treatment group. The data summaries are prepared by the DMC from information provided by the PI.

Closed Sessions: These sessions will only be attended primarily by DMC voting, advisory and staff members who will discuss confidential data from the study, including information on efficacy and safety by treatment arm, if necessary. The DMC may decide whether to remain blinded to treatment assignments during closed meetings. If the closed session occurs on a conference call, steps will be taken to ensure that only the appropriate participants are on the call and to invite others to re-join the call only at the conclusion of the closed session. The PI will not attend closed sessions. The study biostatistician may, at the request of committee members, attend closed sessions when confidential study data is needed in order to evaluate study safety.

Minutes for each meeting will be recorded by CSR&D Program Staff who will work with the DMC Administrator and DMC Managers to ensure that they are promptly reviewed by the DMC Chairperson and approved by the Acting Director of CSR&D. Minutes of the open sessions will be made available to the study team and the IRB(s) of record.

8. Reporting Requirements for the DMC

Proper minutes will be collected at each DMC meeting to ensure that there is a physical record of any and all decisions and recommendations. The required documentation for DMC meetings for the study includes the following:

- Identification of DMC members available for the meeting
- Identification of DMC administrative personnel available for the meeting
- Identification of excused personnel
- Notification of changes in membership and next scheduled DMC meeting
- Review of study data
- Review of study summary report
- Committee recommendations and approvals

CSR&D CO Program Staff will be responsible for the preparation of committee minutes that address the above. Minutes must then be reviewed for accuracy in collaboration with the DMC Administrator and Manager and then endorsed by the DMC Chairperson before being submitted to Acting Director, CSR&D, for final approval. Meeting minutes, endorsed by the committee Chairperson, must be submitted to the Acting Director, CSR&D, within a timely manner, typically 10 working days following a convened DMC meeting.

Before giving final approval, the Acting Director, CSR&D, as sponsor of the research, may decide to contact the PI directly, without DMC Office involvement, to discuss with the PI recommendations made during the DMC meeting. A copy of the approved minutes, reflecting the committee's review, and the Acting Director, CSR&D's requests or recommendations, is then furnished to the PI and the VA facility Associate Chief of Staff/Research.

Once minutes have been endorsed by the Acting Director, CSR&D, they are considered final and archived with other DMC documentation. Investigators receive copies of signed and approved minutes from the DMC for open sessions only while the study is active.

9. Reports of DMC Proceedings

Open reports are available to all who attend the open session of the DMC. Contents of open reports may contain the following:

- Statistical commentary explaining issues presented in Open Report figures, tables and accrual target graphs
- DMC monitoring plan and summary of Open Report data presented at prior DMC meetings
- Major protocol changes
- Information on patient screening
- Study accrual by month by institution
- Eligibility violations
- Quality control variables
- Baseline characteristics
    - Demographics
    - Laboratory values and other measurements
- Length of follow-up

- Protocol deviations, especially deviations from the assigned treatment plan
- AE data summaries
- SAE reports and aggregate SAE data summaries
- Withdrawals from treatment and withdrawals from study.

Closed reports are made available by request and only to those attending closed sessions of the DMC. Closed reports are prepared by the primary trial biostatistician or designee. A basic principle of research integrity is that anyone directly involved in the collection of the research data should not have knowledge of treatment comparisons until data collection is complete. This reduces the likelihood of bias. Thus the study PI and others directly involved in collection of study data and evaluation of study patients will not have access to closed reports or any other treatment comparison summaries. Contents of closed reports may include the following:

- Detailed statistical commentary explaining issues raised by Closed Report figures and tables (by treatment group)
- DMC monitoring plan and summary of Closed Report data presented at prior DMC meetings
- Repeat of the Open Report information, in greater detail by treatment group
- Analysis of primary and secondary efficacy endpoints by treatment group.

If the DMC has elected to receive closed reports where treatment group identification is masked, the primary study biostatistician will unmask the treatment groups upon request by the DMC.

Department of Veteran Affairs

Clinical Sciences Research & Development (CSR&D)

Data Monitoring Committee (DMC) Charter Signature Page

I, **Michael Hollifield, M.D.**, Principal Investigator for the "Acupuncture for PTSD in Combat Veterans," accepted by the Chair on behalf of all members of the DMC, agree to the terms outlined in this Charter 1.0. Any changes to the terms outlined in this Charter will be communicated by the Chair to committee members; consent of committee members, after review, to any changes to this Charter must be obtained by the Data Monitoring Committee Manager.

7/12/2016

Michael Hollifield, M.D.
Signature, Principal Investigator

Date

Digitally signed by VHANYNWolkiA
Date: 2016.07.18 09:02:18 -04'00'

Adam Wolkin, M.D., Chair
Committee Chair Signature

Date

7-28-16

Theresa Gleason, Ph.D.,
Acting Director
Clinical Science Research & Development Service
Department of Veterans Affairs

Date

Appendix

Table 1

Name of Member Reviewing	Role on DMC	Responsibilities
**Adam Wolkin, M.D.**	Chair of DMC	Moderates meetings for both initial review and ongoing study reports
**Vishal Kamani, M.D.**	Ad hoc member	Primary Reviewer of protocol and ongoing reports.
**Tara Galovski, Ph.D.**	Voting Member	Secondary Reviewer for protocol and ongoing reports
**Ping Luo, Ph.D.**	Voting Member	Biostatistical Reviewer for protocol and ongoing reports

Figure 1

Organizational Chart

The following diagram illustrates the organizational relationship between this CSR&D DMC and the stakeholders in the study.

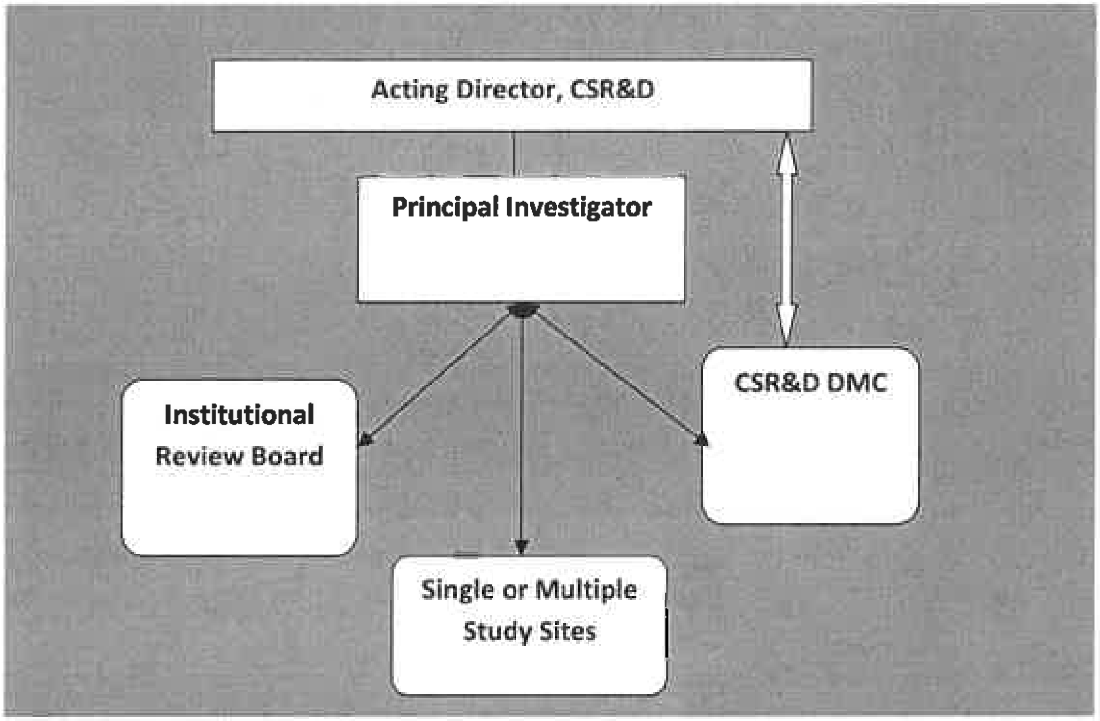

Table 2

**Meeting / Review Type**	**Scheduled Time**	**Purpose**	**Required Attendees**
Initial meeting	Prior to enrollment of study participants (5/12/16)	• Review charter template and draft in study specific information<br>• Identify data for review at future meetings, and presentation and format data will be presented<br>• Review protocol including safety monitoring and statistical analysis plan<br>• Study team will discuss with DMC a statistical model that will be used in review and data monitoring	All DMC Members
Regularly scheduled DMC meetings	Progress/enrollment reports every 4 months	Progress reports for study status, AEs and SAE summary, enrollment/randomization and withdrawal figures.	
Ad hoc conference calls			
Review of formal interim data analyses			

(if applicable)			
(Add other necessary meetings)			

Recommendations from the DMC Review of the Study, pertaining to safety or data integrity, were as follows:

Procedures for Serious Adverse Event reporting throughout the study were briefly discussed. No safety issues were identified.

Safety Plan for Study **Date/Version**

6/23/16 V. 1.0

Adverse Events: Defined as "any untoward occurrence (physical, psychological, social or economic) in a human subject participating in research" (ORO Handbook 1058.01).

Minimizing Risks: The research team will follow guidelines for ethically sound research. Other protections are:

Data Control: Confidentiality protections will be strongly upheld. There will be identifying numbers (ID) and not names of participants on all documents. All data will be kept in locked cabinets at the VALB, and documents that have both ID numbers and names on them (such as the master list of participants) will be kept separate from those with names on them (such as consent forms), which will be kept separate from documents with ID numbers on them (such as assessment forms). Data will be managed as described above.

Protection of Participants

Recruitment and informed consent:
The methods of recruitment, informed consent, interviewing, and other assessment is intended to be protective for the participants, as they are potentially vulnerable to exploitation and harm given their medical/psychiatric status. We are attentive to ethical protection by: 1) following guidelines for ethically sound research, and 2) by having the capacity at the VA Hospital to review research forms and treatment protocols from both a scientific and ethical perspective. Finally, we will encourage participants to ask questions and include family and friends in the informed consent decision process. Participants will be assessed for their decisional capacity for informed consent, making treatment decisions, and having continued assent to participate in the trial. Participants will know that this is totally voluntary, is a developing treatment option, and that there are other, more proven treatment options available to them.

Urgent treatment needs:
We recognize the variability of individual responses to answering questionnaires and interview questions related to PTSD, other life events, symptoms, and current health and social status. Team members will be sensitive and helpful to any individual participant who is distressed, whether or not the distress is from a research effect. The research assistant (RA) will initially field all calls and inquiries from participants

about urgent treatment needs. If it is PTSD or study related, the RA will immediately troubleshoot the potential problem with the PI. If it is emergent, the RA will help the participant make a plan for a visit, which may include calling emergency transportation.

All research staff has or will receive training about the possible effects of hearing about PTSD experiences and distressing symptoms. This training will include identifying a therapist or therapy group that will be willing to see any staff member at his/her own cost during the research project.

Adverse Events:
Clinical Interviews

It is possible that some participants may find answering questions and discussing their symptoms and traumatic experience distressing. All clinicians are trained to address any distress that may occur. You have the right to discontinue the interview at any time.

Psychophysiological assessment

Each of the tests involved in collecting biological measures such as heart rate, blood pressure, and perspiration is a standard, non-painful and non-invasive procedure and used widely in clinical research and practice. There is no medical risk associated with these measures. During part of the psychophysiological assessment, a puff of air will be blown at your neck at random intervals. The puff of air is similar to going to the eye doctor and a puff of air is blown in your air to check for glaucoma. Due to the startling nature of the air puff this may be distressing to some patients. You have the right to discontinue the assessment at any time. Study doctors will be available to address any distress that may occur.

Acupuncture

It is possible that some participants may experience minor bruising, hematoma and bleeding, fainting or nausea while receiving acupuncture. There is a less than 1 in 10,000 risk of pneumothorax (collapsed lung), this can occur when air leaks into the space between the lung and chest wall. The incidence of this occurring has been reported in cases where inexperienced clinicians performed acupuncture. Other adverse events such as organ puncture, infection, physiological or psychological responses have an incidence rate of 1 in 10,000 to 1 in 100,000. To reduce the risk of these occurrences, only licensed, trained, and experienced clinicians will be performing acupuncture.

Investigators will review potential side effects with you at each visit in order to monitor any occurrence of these symptoms. If you develop symptoms of psychosis, or intent to commit suicide, homicide, or other violent acts; or are judged by the researchers to have become severely disabled in some other way, you will have to discontinue the study, but will first be provided with appropriate emergency care and treatment referrals.

Individual AE reports need not be provided to the DMC. Rather, an AE summary table should be provided to the DMC as part of each 4-month progress report. Following is the table structure to be used:

	Treatment Group 1	Treatment Group 2

	(state number randomized)			
**General Adverse Event Descriptor**	**# of individual reports**	**# of patients reporting at least once**	**Treatment group, if study not blinded**	**Care Provided**
**e.g. alteration of blood pressure**				

Following receipt of the summary table, the DMC may request additional information on specific AE reports.

<u>Serious Adverse Events</u>: Any of the following qualify as a SAE reportable to the DMC:

1. **Death**
2. **New or prolonged hospitalization**
3. **Life threatening (imminent risk of death)**
4. **Results in persistent significant disability/incapacity**
5. **Results in congenital anomaly/birth defect**
6. **Any other condition that may jeopardize the participant or others in relation to the participant, requiring immediate action, whether medical, surgical or psychological, to be taken with the research subject to prevent further harm, or one of the above outcomes.**

These events should be reported <u>regardless of any differences in reporting requirements for the local IRB</u>. If the event itself meets the definition of an SAE, it should be reported to the DMC, whether or not it is potentially related to study procedures or whether the events are anticipated or unanticipated. (Details of how to transmit SAE information, and the SAE form to use for DMC filing, are attached.)

In addition to the requirement to report individual SAEs within 48 hours to the DMC, a condensed listing of all SAEs should be included with progress report. The following format should be used:

**Type of SAE**	**Date of onset**	**Date of resolution**	**Treatment group-if study not blinded**	**Relatedness to study participation or study treatment?**	**Care provided, adjustments to study treatments**
**e.g., hospitalization**					
